# Genome-Wide Identification and Expression Profiling of the GATA Gene Family Reveals Candidate Genes Associated with GA_3_-Induced Shoot Elongation in *Citrus grandis* ‘Wanbai’

**DOI:** 10.3390/ijms27198542

**Published:** 2026-09-24

**Authors:** Chenxiang Ma, Yuanyuan Xu, Jian Han, Youhan Kong, Liying Yang, Di Feng, Hongyan Fu, Syed Bilal Hussain, Peng Chen, Lingxia Guo

**Affiliations:** 1 College of Forestry, Central South University of Forestry and Technology, Changsha 410004, China; 2 Yuelushan Laboratory, Changsha 410125, China; 3 Institute of Horticultural Research, Hunan Academy of Agricultural Sciences, Changsha 410125, China; 4 School of Agriculture and Food Science, University College Dublin, D04V1W8 Dublin, Ireland

**Keywords:** pummelo, zinc-finger proteins, cis-acting elements, phylogenetic analysis, hormone-associated expression, tree architecture

## Abstract

Plant GATA transcription factors are zinc-finger proteins involved in signal transduction, growth and development. However, their potential involvement in plant height and gibberellin-associated shoot growth in pummelo insufficiently characterised. In this study, members of the *CgGATA* gene family were identified genome-wide and characterised through analyses of physicochemical properties, chromosomal distribution, phylogenetic relationships, gene structures, conserved motifs, collinearity and predicted cis-acting elements in putative upstream regulatory regions. RT-qPCR was used to examine expression profiles across sampled tissues, and expression changes associated with prolonged gibberellin (GA_3_) treatment in ‘Wanbai Pummelo’ shoot tips, and expression patterns in tall and dwarf pummelo genotypes. A total of 23 *CgGATA* genes were identified and classified into four phylogenetic subfamilies. Members of the same subfamily generally shared similar gene structures and conserved-motif compositions. Comparative collinearity analysis identified more syntenic GATA gene pairs between pummelo and sweet orange than between pummelo and *Arabidopsis thaliana*. The putative upstream regulatory regions contained predicted elements associated with light, phytohormone, growth and stress responses. The genes exhibited distinct expression profiles across the sampled tissues, with many showing relatively high within-gene values in stems and leaves. Prolonged GA_3_ treatment promoted seedling shoot elongation and was associated with changes in the transcript abundance of several *CgGATA* genes at 28 d. Integration of these expression profiles, the tall dwarf comparison and predicted gibberellin-responsive elements prioritised *CgGATA11* and *CgGATA18* as candidates for investigating plant height variation and shoot elongation in pummelo. These findings provide a basis for functional studies but do not establish that either gene directly regulates gibberellin signalling or plant height.

## 1. Introduction

Pummelo (*Citrus grandis*, synonym *Citrus maxima*) is an economically important fruit tree in the family Rutaceae. Citrus tree architecture influences planting density, canopy light distribution and the efficiency of pruning, spraying and harvesting; consequently, compact or dwarfing phenotypes are valuable for intensive orchard systems [1]. Recent work has identified several regulators of citrus architecture, including *CsTAC1*, which affects branch angle and endogenous hormone profiles, a BBX22-HY5 transcriptional cascade associated with plant height, and a miR156-SPL module that modulates gibberellin biosynthesis and plant height [2,3,4]. Identifying additional genes associated with shoot elongation could therefore support the development of pummelo germplasm with improved tree architecture.

Plant height is controlled by genetic, developmental, and environmental inputs, including crosstalk among gibberellins, auxins, brassinosteroids, and strigolactones [5,6]. Gibberellins are particularly important regulators of cell division, cell elongation, and stem growth [7,8,9]. Plant GATA transcription factors are type-IV zinc-finger DNA-binding proteins, typically characterised by a C-X_2_-C-X_18_–_20_-C-X_2_-C motif followed by a basic region [10,11]. Members of this family participate in photomorphogenesis, chlorophyll biosynthesis, chloroplast development, root and leaf development, flowering, germination, and responses to environmental signals [10,11]. Transcriptomic analysis of graphene oxide-treated buckwheat roots also identified differentially expressed GATA genes, along with changes in transporter and reactive oxygen species detoxification pathways, suggesting a possible association with stress-related detoxification responses [12]. In *Arabidopsis thaliana*, the GATA factors GNC and GNL/CGA1 act downstream of DELLA proteins and PHYTOCHROME-INTERACTING FACTORS to repress gibberellin-promoted growth [13].

Genome-wide GATA-family analyses have been reported in *A. thaliana*, rice, apple, several Rosaceae species, sweet orange, and poplar [10,14,15,16,17,18]. These studies indicate conservation of the GATA zinc-finger domain together with diversification in gene structure, regulatory sequences, and expression. Functional analyses also link individual GATA factors to shoot development. For example, rice NECK LEAF 1 influences reproductive organogenesis and upper-internode elongation; *Arabidopsis* GNC and GNL/CGA1 restrict gibberellin-mediated growth; and overexpression of *PdeGATA3* produces a dwarf phenotype in poplar, accompanied by changes in the expression of genes related to meristem activity and gibberellin metabolism [13,19,20]. However, the pummelo GATA family remains insufficiently characterised in relation to prolonged GA_3_ treatment, shoot growth and contrasting plant-height phenotypes.

In this study, members of the *CgGATA* family were identified in the pummelo reference genome and characterised by analysing their chromosomal distribution, phylogenetic relationships, gene structures, conserved motifs, collinearity, and predicted cis-acting elements in putative upstream regulatory regions. RT-qPCR was used to examine expression profiles across the sampled tissues. Expression changes associated with prolonged GA_3_ treatment were evaluated in ‘Wanbai Pummelo’ shoot tips, and expression was compared between tall ‘Shatian Pummelo’ and dwarf ‘Aiwan Pummelo’ at two spring-shoot developmental stages. Integrating these expression profiles with the cis-element predictions enabled the prioritisation of candidate *CgGATA* genes for further investigation of plant height variation and shoot elongation. Throughout the manuscript, italicised gene symbols denote genes or transcripts, whereas the corresponding non-italicised symbols denote their encoded proteins. The term ‘putative upstream regulatory regions’ refers to sequences upstream of annotated translation start codons, rather than experimentally validated promoters.

## 2. Results

### 2.1. Identification, Physicochemical Properties, and Chromosomal Distribution of CgGATA Genes

A total of 23 putative *GATA* loci were identified in the pummelo reference genome and designated *CgGATA1*–*CgGATA23* according to their chromosomal positions (Supplementary Table S1). The predicted properties of the encoded proteins are summarised in Table 1. Protein length ranged from 103 amino acids (CgGATA5) to 542 amino acids (CgGATA13), and predicted molecular mass ranged from 11.55 to 60.30 kDa. The theoretical isoelectric points ranged from 4.68 (CgGATA7) to 10.30 (CgGATA16); eleven of the 23 proteins had predicted pI values above 7.0.

The predicted instability indices ranged from 34.51 to 67.07. CgGATA14 had an index below 40 and was therefore predicted to be stable, whereas the other 22 proteins had indices above 40. Aliphatic indices ranged from 45.63 to 69.50. All proteins had negative grand average of hydropathicity (GRAVY) values (−1.275 to −0.452), consistent with predicted hydrophilicity, and all were predicted to localise to the nucleus.

Twenty-two *CgGATA* genes mapped to the nine assembled chromosomes, whereas *CgGATA23* was located on an unanchored sequence designated ChrUn (Figure 1). Chromosome 5 contained the most *CgGATA* genes, with five members (*CgGATA13*–*CgGATA17*). Chromosomes 1 and 4 each contained four genes; chromosomes 2, 3, and 8 each contained two; and chromosomes 6, 7, and 9 each contained one. Several loci were closely spaced on chromosomes 2, 3 and 4, but their proximity alone does not establish tandem duplication.

### 2.2. Phylogenetic Relationships Among GATA Proteins

To examine evolutionary relationships, a neighbour-joining tree was constructed from full-length GATA protein sequences from pummelo, sweet orange, and *A. thaliana* (Figure 2). The proteins formed four major subfamilies, designated I–IV. Of the 23 pummelo proteins, 10 belonged to Subfamily I, eight to Subfamily II, four to Subfamily III, and one, CgGATA13, to Subfamily IV. Each subfamily contained representatives of all three species, a pattern consistent with the establishment of the major GATA lineages before their divergence. Several pummelo proteins clustered closely with sweet orange homologues. The phylogenetic groupings were also broadly consistent with similarities in gene structure and motif composition.

### 2.3. Gene Structure and Conserved-Motif Composition of the CgGATA Family

The exon–intron organisation of the 23 *CgGATA* genes and the conserved-motif composition of their encoded proteins were examined alongside the phylogenetic classification (Figure 3). Fourteen conserved motifs were identified in the CgGATA proteins (Figure 3B), and their sequence logos are presented in Figure 4. Motif 1 corresponds to the conserved GATA zinc-finger domain and was detected in all 23 CgGATA proteins. Members of the same subfamily generally displayed similar motif compositions and arrangements. Subfamily I proteins commonly contained Motifs 2, 4, 8, and 9, whereas Subfamily II proteins generally contained fewer motifs. Motifs 5 and 14 occurred predominantly in Subfamily III. CgGATA13, the sole Subfamily IV member, contained only Motif 1 under the parameters used. Genes in Subfamilies I and II generally contained fewer introns than those in Subfamily III, whose members had comparatively complex exon–intron structures. Annotated 5′- and 3′-untranslated regions were present in several genes. Overall, genes within a subfamily tended to share structural features, although variation was evident among individual members.

### 2.4. Intraspecific and Interspecific Collinearity of CgGATA Genes

Intraspecific collinearity analysis was used to examine genomic relationships among *CgGATA* loci (Figure 5A). Collinear relationships involving *CgGATA* genes were detected among chromosomes 1, 4, 5, 7, 8, and 9. No *CgGATA* collinear pairs were detected on the remaining chromosomes under the parameters used.

Interspecific analyses identified 19 syntenic *GATA* gene pairs between pummelo and *A. thaliana* and 28 between pummelo and sweet orange (Figure 5B). The larger number detected in the pummelo–sweet orange comparison is consistent with the closer relationship between the two citrus species. Several *CgGATA* genes corresponded to multiple syntenic loci in a comparative genome, suggesting lineage-specific duplication and/or differential gene retention.

### 2.5. Predicted Cis-Acting Elements in the Putative Upstream Regulatory Regions of CgGATA Genes

Putative cis-acting regulatory elements were predicted within the 2000 bp sequences upstream of the annotated start codons of the 23 *CgGATA* genes (Figure 6). Twenty-eight element types were identified: 11 associated with light responses, nine with phytohormone responses, three with growth and development, and five with stress. ACE, Box 4, and G-box elements were widely distributed. Hormone-associated predictions included elements linked to responses to abscisic acid, methyl jasmonate, salicylic acid, gibberellin and auxin, whereas stress-associated predictions included anaerobic- and low-temperature-responsive elements and MYB-binding sites. Predicted gibberellin-responsive elements were found in the putative upstream regulatory regions of *CgGATA4*, *CgGATA5*, *CgGATA8*, *CgGATA9*, *CgGATA10*, *CgGATA11*, *CgGATA12*, *CgGATA13*, *CgGATA15*, *CgGATA17*, *CgGATA18*, *CgGATA21,* and *CgGATA22*.

### 2.6. Expression Profiles of CgGATA Genes Across Sampled Tissues and in Contrasting Plant-Height Genotypes

RT-qPCR was used to examine the relative transcript abundance of the 23 *CgGATA* genes in roots, stems, leaves, floral tissue, fruits, and seeds of ‘Wanbai Pummelo’ (Figure 7A). After row-wise scaling, the genes displayed distinct within-gene expression patterns across the sampled tissues. Many genes had their highest or near-highest relative values in vegetative tissues, particularly stems and leaves, whereas several showed maxima in fruits or seeds. *CgGATA3*, *CgGATA9* and *CgGATA21* had relatively high values in leaves; *CgGATA16* in seeds; and *CgGATA11* in fruits. These patterns describe variation across the sampled tissues but cannot separate tissue effects from differences in plant age, developmental stage or sampling season.

Expression was subsequently compared in spring-shoot tips of tall ‘Shatian Pummelo’ and dwarf ‘Aiwan Pummelo’ at two developmental stages (Figure 7B). The mature trees had mean heights of approximately 3.60 and 1.20 m, respectively (Figure S1). To statistically evaluate the tall-versus-dwarf (ST-versus-AW) comparison rather than relying on a descriptive reading of the heatmap, we applied a two-way ANOVA with genotype, developmental stage, and their interaction (genotype × stage) as factors. The genotype × stage interaction was significant for all four gene blocks analysed (GATA1–6: F(15, 36) = 77.69, *p* < 0.0001; GATA7–12: F(15, 36) = 879.2, *p* < 0.0001; GATA13–18: F(15, 36) = 229.8, *p* < 0.0001; GATA19–23: F(12, 30) = 26.52, *p* < 0.0001). The main effects of genotype and developmental stage were also significant across all four blocks (*p* < 0.0001). The interaction terms accounted for a large proportion of the total variation (39.13–65.74%), confirming that the ST-versus-AW difference in *CgGATA* expression is dependent on developmental stage. At S1, *CgGATA1*, *CgGATA2*, *CgGATA3*, *CgGATA6*, *CgGATA7*, *CgGATA9*, *CgGATA11*, *CgGATA13*, *CgGATA14*, *CgGATA15,* and *CgGATA18* displayed higher row-scaled expression values in ‘Shatian Pummelo’ than in ‘Aiwan Pummelo’. At S2, *CgGATA5*, *CgGATA6*, *CgGATA10*, *CgGATA12*, *CgGATA17*, *CgGATA20* and *CgGATA21* displayed higher values in ‘Aiwan Pummelo’, whereas *CgGATA22* and *CgGATA23* were relatively high in both genotypes. In the heatmap, *CgGATA11* and *CgGATA18* had higher values in the tall genotype at both stages. These heatmap observations are descriptive and do not, by themselves, establish statistically significant genotype differences.

### 2.7. Effects of Prolonged GA_3_ Treatment on Seedling Height and CgGATA Expression

Three-month-old ‘Wanbai Pummelo’ seedlings were sprayed with 50 mg L^−1^ GA_3_ or the corresponding control solution. By 28 d, GA_3_-treated seedlings showed visibly greater shoot elongation (Figure 8A). Linear mixed-effects analysis identified significant main effects of treatment (*p* < 0.05) and time (*p* < 0.001), as well as a significant treatment × time interaction (*p* < 0.001), indicating that the effect of GA_3_ on plant height varied over time. Post hoc comparisons revealed no significant difference in plant height between the control and GA_3_-treated groups at 0, 7, 14, or 21 d, whereas GA_3_-treated seedlings were significantly taller at 28 d (Figure 8B). Mean height at 28 d was 6.65 ± 1.18 cm in the control group and 10.04 ± 1.71 cm in the GA_3_-treated group.

Shoot-tip expression was assessed at 28 d following repeated GA_3_ application (Figure 8C,D). Eleven genes—*CgGATA1*, *CgGATA3*, *CgGATA4*, *CgGATA7*, *CgGATA11*, *CgGATA12*, *CgGATA14*, *CgGATA15*, *CgGATA17*, *CgGATA18* and *CgGATA21*—had significantly higher relative transcript abundance in the GA_3_-treated group. Four genes, *CgGATA2*, *CgGATA6*, *CgGATA9,* and *CgGATA20*, had significantly lower abundance. The remaining eight genes—*CgGATA5*, *CgGATA8*, *CgGATA10*, *CgGATA13*, *CgGATA16*, *CgGATA19*, *CgGATA22* and *CgGATA23*—did not show statistically significant differences under the conditions tested. Thus, 15 of the 23 family members showed expression changes associated with prolonged GA_3_ treatment at this sampling time.

### 2.8. Prioritisation of CgGATA Candidates for Studies of Shoot Elongation and Plant Height Variation

Candidate genes were prioritised by integrating four qualitative sources of information: expression in vegetative or shoot-tip tissues; expression changes associated with prolonged GA_3_ treatment; contrasting expression patterns between tall and dwarf pummelo genotypes; and predicted gibberellin-responsive elements in putative upstream regulatory regions. *CgGATA11* and *CgGATA18* were selected for further investigation because both showed increased transcript abundance after prolonged GA_3_ treatment, higher row-scaled values in the tall genotype at S1 and S2, and predicted GARE motifs in their 2000 bp upstream regions. Supplementary Table S2 lists the motif type, nucleotide sequence, reported position, length, strand and functional annotation of these predicted sites. The predictions do not establish that these regions mediate gibberellin-dependent transcription. Similarly, differences in qualitative heatmaps do not constitute statistical evidence of a genotype effect. This prioritisation therefore identifies hypotheses for functional testing rather than establishing a causal role for either gene in shoot elongation or plant-height variation.

## 3. Discussion

GATA transcription factors are evolutionarily conserved zinc-finger proteins with diverse roles in plant development and signalling [10,11]. We identified 23 *CgGATA* genes and classified them into four phylogenetic subfamilies. Members of each subfamily generally shared exon–intron structures and motif compositions, whereas differences were more pronounced among subfamilies. Comparable subfamily-associated conservation has been described in apple, Rosaceae species, sweet orange, and poplar [15,16,17,18].

Representatives of pummelo, sweet orange, and *A. thaliana* occurred in each of the four subfamilies, and several pummelo proteins clustered closely with sweet orange homologues. More GATA syntenic pairs were detected between pummelo and sweet orange than between pummelo and *A. thaliana*, consistent with their relative phylogenetic distances. However, detected synteny counts can also be influenced by assembly quality, annotation completeness, and analysis parameters. Moreover, phylogenetic proximity alone does not establish functional equivalence, because homologues can diverge in expression and regulatory activity.

The putative upstream regulatory regions of *CgGATA* genes contained predicted cis-elements associated with light, phytohormones, development, and stress. Light-responsive elements are consistent with established roles of plant GATA factors in photomorphogenesis, chlorophyll biosynthesis and chloroplast function [10,11]. Predicted elements associated with gibberellin, auxin, abscisic acid, methyl jasmonate, and salicylic acid suggest potential hormonal regulation, and similar combinations have been reported in the GATA upstream regions of Rosaceae, sweet orange, and poplar [16,17,18]. Nevertheless, the presence of a short sequence motif does not, by itself, demonstrate regulatory activity. Experimental assays are required to establish whether the predicted elements are functional, and variation in cis-regulatory sequence can have context-dependent effects on gene expression [21].

The *CgGATA* genes exhibited distinct expression profiles across the sampled tissues. Many reached within-gene maxima in stems or leaves, whereas several were relatively high in fruits or seeds. Variation in GATA expression among tissues has also been reported in Chinese pear, sweet orange, and poplar [16,17,18]. In the present study, row-wise z-score scaling visualised the expression pattern of each gene across samples; it did not permit absolute comparison of transcript abundance between genes or establish statistical significance. Interpretation is also constrained by the sampling design: vegetative tissues were collected from seedlings, whereas reproductive tissues were collected from mature trees at different times of year. Tissue identity was therefore confounded with plant age, developmental stage and sampling season. These profiles provide descriptive information to select genes for further study, but they do not establish tissue- or stage-specific regulatory functions.

Gibberellins regulate cell division, cell elongation, shoot growth, and plant height [7,8]. Prolonged GA_3_ treatment increased ‘Wanbai Pummelo’ seedling height at 28 d. At the same time point, 11 *CgGATA* genes showed higher transcript abundance, four showed lower abundance, and eight showed no statistically significant difference. These patterns indicate variation among family members in their expression associations with prolonged GA_3_ treatment. Because expression was measured only at day 28 following repeated applications, the experiment cannot distinguish direct transcriptional responses to gibberellin from secondary changes associated with development, tissue composition or feedback regulation. Early-time-course experiments would be needed to resolve these possibilities.

Functional links between GATA factors and gibberellin pathways have been established in other species. In *A. thaliana*, GNC and GNL/CGA1 act downstream of DELLA proteins and PHYTOCHROME-INTERACTING FACTORS to restrict gibberellin-promoted growth [13]. The DELLA protein RGL2 interacts with the DOF transcription factor DOF6 to promote *GATA12* expression during the establishment of primary seed dormancy [22]. In poplar, overexpression of *PdeGATA3* causes dwarfism and alters the expression of genes associated with meristem activity and gibberellin metabolism [20]. These studies provide a rationale for investigating *CgGATA* genes whose expression changes during prolonged GA3 treatment. However, the present results do not establish that these genes act directly downstream of DELLA proteins or regulate gibberellin metabolism. Unlike the cited functional studies, this pummelo study prioritises candidates from bioinformatic and expression evidence without establishing their regulatory mechanisms.

Expression comparisons between tall ‘Shatian Pummelo’ and dwarf ‘Aiwan Pummelo’ provided an additional, non-causal source of information. Several genes had higher row-scaled values in the tall genotype at S1, whereas a different subset had higher values in the dwarf genotype at S2. The two-way ANOVA results provide formal statistical support for the stage-dependent differences in *CgGATA* expression between ST and AW. For all four gene blocks, the genotype × stage interaction was highly significant and accounted for a large proportion of the total variation (up to 65.74%). This indicates that the ST-versus-AW contrast is not constant across development. This finding justifies reporting stage-resolved contrasts rather than interpreting the heatmap descriptively. These patterns may reflect genotype, developmental stage, or their interaction. Because the cultivars differ in genetic background and plant height, the differences in expression cannot be attributed solely to height variation. The heatmap provides a descriptive comparison, but gene-wise analysis of the biological-replicate data is required to establish which same-stage genotype differences are statistically supported. These observations should not be interpreted as evidence that higher transcript abundance causes the taller phenotype.

Integrating the expression profiles and predicted upstream elements prioritised *CgGATA11* and *CgGATA18* for further investigation. Both showed higher transcript abundance after prolonged GA_3_ treatment, higher row-scaled values in the tall genotype at both stages, and predicted GARE motifs. Neither the direction of an expression change nor a motif prediction establishes whether the encoded factor promotes or restricts elongation. In particular, the data do not establish a DELLA-*CgGATA* regulatory pathway, in contrast to studies that directly investigate DELLA activity [23]. Genetic perturbation, experimental assessment of subcellular localisation, DNA-binding, and promoter-reporter, and measurements of endogenous gibberellins would help determine whether these candidates contribute to shoot growth and how they act.

Overall, this study provides a genome-wide description of the pummelo *GATA* family and combines upstream cis-element prediction, expression profiling across sampled tissues, a GA_3_-associated growth phenotype, and expression comparisons between contrasting genotypes. Interpretation is constrained by the predictive nature of the bioinformatic analyses, confounding from tissue sampling, genetic differences between cultivars, a single expression time point after prolonged GA3 treatment, and the absence of direct functional validation. Accordingly, the proposed involvement of *CgGATA11* and *CgGATA18* in shoot elongation and plant height variation remains a working hypothesis. Genetic, biochemical, and physiological analyses are needed to establish whether and how these candidates contribute to citrus tree architecture.

## 4. Materials and Methods

### 4.1. Plant Materials and Growth Conditions

Seeds of ‘Wanbai Pummelo’ were obtained from the pummelo germplasm nursery of the Institute of Horticultural Research, Hunan Academy of Agricultural Sciences, Changsha, China. Seeds were sown in a 3:1:2 (*v*/*v*/*v*) mixture of peat, perlite, and garden soil. Seedlings were maintained in a controlled-environment chamber at 25 °C, with a 16 h light/8 h dark photoperiod and 70% relative humidity.

Uniform, healthy three-month-old seedlings without visible disease or pest damage were selected for GA_3_ treatment. For expression profiling across sampled tissues, roots, stems, and leaves were collected from uniform four-month-old ‘Wanbai Pummelo’ seedlings. Flowers were sampled in April 2025, and mature fruits and seeds were harvested in November 2025 from field-grown mature trees. Samples from mature trees were collected from the east, south, west, and north aspects of the outer canopy; damaged or diseased tissues were excluded. Three biological replicates were prepared for each tissue type, each comprising equal quantities of tissue pooled from three independent seedlings or mature trees. Samples were immediately frozen in liquid nitrogen and stored at −80 °C until RNA extraction. Because plant age, developmental stage, and sampling season varied across tissue types, the analysis describes expression profiles across the sampled tissues rather than isolating the effect of tissue identity. Tall ‘Shatian Pummelo’ and dwarf ‘Aiwan Pummelo’ were used as contrasting plant-height genotypes. Twelve-year-old trees were grown in the same germplasm orchard under standardised management. Average mature-tree height was approximately 3.60 m for ‘Shatian Pummelo’ and 1.20 m for ‘Aiwan Pummelo’. Height was measured in three trees per genotype as the vertical distance from the soil surface at the root-shoot junction to the apical point of the main shoot.

Shoot apex samples from the tall and dwarf pummelo genotypes were collected at two spring-shoot developmental stages, designated S1 and S2. S1, the rapid elongation stage, comprised shoots with 3 fully unfolded leaves, 2 to 3 visible internodes, a shoot length of 4–6 cm, and active internode elongation. S2, the shoot maturation stage, comprised shoots from the same cohort with five to six fully expanded leaves, four to five internodes, and cessation of internode elongation. At each stage, shoots meeting these morphological criteria were collected from both genotypes on the sampling date. Three biological replicates were prepared for each genotype × stage combination. Each replicate comprised tissue from multiple shoots; the tissue was immediately frozen in liquid nitrogen and stored at −80 °C.

### 4.2. Exogenous GA_3_ Treatment and Plant-Height Measurement

Seedlings were assigned to a control treatment and a treatment containing 50 mg L^−1^ gibberellic acid (GA_3_). To prepare the GA_3_ solution, 25 mg of GA_3_ powder was dissolved in 200 μL of 1 mol L^−1^ sodium hydroxide solution, and diluted to a final volume of 500 mL with sterile water. The control solution contained the same amount of sodium hydroxide as the solvent treatment. Both solutions were supplemented with 0.2% (*v*/*v*) Tween-20 to improve spray adhesion to leaf surfaces. Each treatment comprised three biological replicates of 10 seedlings (30 seedlings per treatment). The biological replicate, defined as a group of 10 seedlings, was the experimental unit for plant height analysis. Spraying began on 23 March 2026 and was repeated at 7-day intervals for four consecutive weeks.

Plant height was recorded immediately before treatment (0 d) and at 7, 14, 21, and 28 d as the distance from the substrate surface to the shoot apex, measured to the nearest 0.1 cm. Shoot-tip samples were collected at 28 d at the same time of day. For each biological replicate, shoot-tip tissues from five seedlings were pooled for RNA extraction. The pooled tissues were immediately frozen in liquid nitrogen and stored at −80 °C.

Longitudinal plant height data were analysed using a linear mixed-effects model implemented in R with the lme4 and emmeans packages. Fixed effects were treatment (control or GA_3_), sampling time (0, 7, 14, 21, and 28 d), and the treatment × time interaction. Biological replicate was included as a random intercept. The mean height of the 10 seedlings within each replicate at each time point was used for modelling, rather than treating individual seedlings as independent treatment replicates. Post hoc pairwise comparisons with Tukey’s adjustment were performed when the interaction was significant. For RT-qPCR expression comparisons, a two-way analysis of variance followed by Tukey’s multiple comparisons test was performed. Statistical significance was set at *p* < 0.05.

### 4.3. Identification of GATA-Family Members

*Arabidopsis thaliana* GATA protein sequences were obtained from The Arabidopsis Information Resource (TAIR, https://www.arabidopsis.org/ accessed on 8 September 2025). A local BLAST TBtools v2.097 search was performed using the Blast Compare Two Seqs module in TBtools v2.097, with an E-value threshold of 1 × 10^−5^. The HMM profile for the GATA domain (PF00320) was retrieved from Pfam (http://pfam.xfam.org/ accessed on 8 September 2025) [24]. The Simple HMM Search module in TBtools was used to screen the *Citrus grandis* proteome for additional candidates. The final workflow used a search-set rule and domain criteria: candidate identifiers from the BLAST and HMM searches were intersected, and only candidates supported by both methods were retained. Redundancy was resolved based on the actual locus rather than on sequence-identity clustering. Of the 91 initial candidates, 33 loci were retained; the remaining 58 single-method candidates were excluded for lacking support from both methods. Retained candidates were further checked against the NCBI Conserved Domain Database (CDD, https://www.ncbi.nlm.nih.gov/Structure/bwrpsb/bwrpsb.cgi accessed on 8 September 2025), and sequences with incomplete conserved domains were excluded from the final candidate set.

### 4.4. Physicochemical Properties and Predicted Subcellular Localisation

Protein length, molecular mass, theoretical pI, instability index, aliphatic index, and grand average of hydropathicity were calculated for each CgGATA protein using ExPASy ProtParam (https://web.expasy.org/protparam/ accessed on 9 September 2025). Subcellular localisation was predicted using Cell-PLoc 2.0 (http://www.csbio.sjtu.edu.cn/bioinf/CellPLoc-2/ accessed on 9 September 2025).

### 4.5. Chromosomal Distribution, Phylogenetic Analysis, Gene Structure, and Conserved Motifs

Chromosomal coordinates were obtained from the annotation of the pummelo reference assembly HWB.v1.0, derived from the ‘Wanbaiyou’ genome [25], and visualised using the Gene Location Visualisation module in TBtools v2.097 [26]. Genes assigned to unanchored scaffolds or contigs were designated accordingly. The HWB.v1.0 and sweet orange SWO.v3.0 genomic, coding-sequence and protein datasets were obtained from the Citrus Pan-genome to Breeding Database (CPBD, http://citrus.hzau.edu.cn/ accessed on 10 September 2025).

GATA protein sequences from pummelo, sweet orange, and *A. thaliana* were obtained from HWB.v1.0, SWO.v3.0, and TAIR10, respectively. Multiple-sequence alignment was performed using ClustalW v2.1 [27]. Alignment columns with missing data were treated by partial deletion using a site-coverage cutoff of 50%, retaining 266 of the 673 alignment positions.

A neighbour-joining tree was constructed in MEGA11 with 1000 bootstrap replicates [28]. The amino acid substitution model was selected using the best-fit model test in MEGA11. The tree was visualised and annotated using iTOL (https://itol.embl.de accessed on 13 September 2026) [29].

Gene structures, including coding sequences, untranslated regions, and introns, were extracted from the pummelo annotation and visualised in TBtools. Conserved protein motifs were identified using MEME Suite v5.5.9 [30], with a maximum of 14 motifs, motif widths of 6–50 amino acids, and default settings for the remaining parameters. Motif composition and gene structure were displayed alongside the phylogenetic tree in TBtools.

### 4.6. Gene Duplication and Collinearity Analyses

A local all-against-all BLASTP search of the pummelo proteome was performed with an E-value threshold of 1 × 10^−5^. Collinear blocks and duplicated gene pairs were identified using the One Step MCScanX module in TBtools v2.097 [26,31]. Intragenomic collinearity was visualised using the Advanced Circos module.

For interspecific analyses, the *A. thaliana* TAIR10 and sweet orange SWO.v3.0 genome assemblies and annotation files were obtained from TAIR and the Citrus Genome Database, respectively. Pairwise collinearity between pummelo and each of the comparison genomes was analysed using the One Step MCScanX module. Relationships involving GATA genes were visualised in TBtools.

### 4.7. Prediction of Cis-Acting Elements in Putative Upstream Regulatory Regions

For each *CgGATA* gene, the 2000 bp sequence upstream of the annotated translation start codon (ATG) was extracted from the *Citrus grandis* HWB.v1.0 genome assembly. Because experimentally verified transcription start sites were unavailable, these sequences were designated putative upstream regulatory regions rather than validated promoters. The sequences were analysed using PlantCARE (https://bioinformatics.psb.ugent.be/webtools/plantcare/html/ accessed on 11 September 2025) to predict cis-acting regulatory elements [32]. Elements associated with light, phytohormones, growth and development, and environmental stress were selected for visualisation using the Basic BioSequence View and HeatMap modules in TBtools.

### 4.8. RNA Extraction, cDNA Synthesis and RT-qPCR

Total RNA was extracted from roots, stems, leaves, floral tissue, fruits, seeds, and shoot tips using the DP672 Plant Total RNA Extraction Kit for polysaccharide- and polyphenol-rich tissues (Tiangen Biotech, Beijing, China), following the manufacturer’s instructions. First-strand cDNA was synthesised from 1 μg of total RNA using the FastKing reverse-transcription kit (catalogue number FP313; Tiangen Biotech). Gene-specific primers were designed using NCBI Primer-BLAST (https://ncbi.nlm.nih.gov/tools/primer-blast/index.cgi accessed on 4 March 2026) [33] and are listed in Supplementary Table S3. *β-Actin* (accession XM_006464503) was used as the reference gene [34]. RT-qPCR was performed using SYBR Green Master Mix on a LightCycler 480 system (Roche, Basel, Switzerland). Each 20 μL reaction contained 1× SYBR Green qPCR Master Mix, 0.2 μM forward primer, 0.2 μM reverse primer, and 100 ng of cDNA template, with nuclease-free water added to bring the reaction to the final volume. The cycling programme comprised 95 °C for 1 min, followed by 40 cycles of 95 °C for 10 s and 60 °C for 20 s. Three biological replicates were analysed per condition, with three technical replicates per biological replicate. Statistical analyses used ΔCt values calculated relative to the reference gene. Two-sided Student’s *t*-tests were used to compare GA_3_-treated and control samples for each gene. The Benjamini–Hochberg false discovery rate (FDR) procedure was used to adjust for multiple testing across the 23 *CgGATA* genes. Relative expression was calculated using the 2^−ΔΔCt^ method for graphical presentation [35]. Heatmaps were generated in TBtools from these relative expression values, which were scaled row-wise. The differential expression analysis and multiple-testing results are included in Supplementary Table S4.

### 4.9. Statistical Analysis

Statistical analyses were performed using R v4.3.1 and GraphPad Prism v8.0.2. Unless otherwise stated, data are presented as the mean ± SE of biological replicates. Longitudinal plant height measurements were analysed using the mixed-effects model described in Section 4.2. The analysis of GA_3_-versus-control expression differences is described in Section 4.8. Statistical significance was defined as *p* < 0.05; the figure annotations use one, two and three asterisks to denote *p* < 0.05, *p* < 0.01, and *p* < 0.001, respectively.

## 5. Conclusions

This study identified 23 *CgGATA* genes in the pummelo reference genome and classified them into four subfamilies. The genes exhibited diverse expression profiles across sampled tissues. Prolonged GA_3_ treatment promoted seedling shoot elongation and was associated with changes in the transcript abundance of 15 genes at 28 d. Integrating the treatment-associated expression patterns, tissue and genotype comparisons, and predicted upstream cis-elements prioritised *CgGATA11* and *CgGATA18* for further investigation. Their proposed involvement in shoot elongation and plant height variation remains a hypothesis rather than a demonstrated regulatory function. These findings provide a basis for the functional characterisation of GATA genes in pummelo shoot growth and tree architecture.

## Figures and Tables

**Figure 1:**
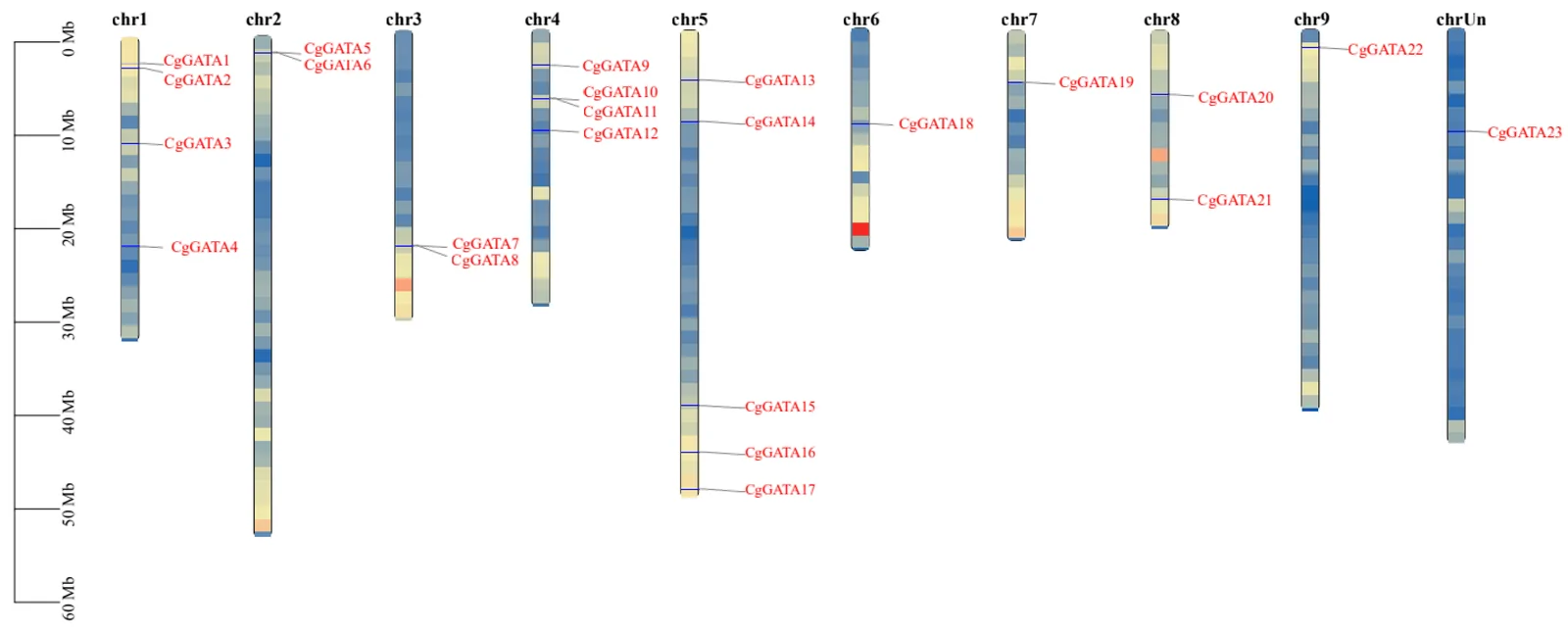
Chromosomal distribution of *CgGATA* genes in the pummelo reference genome. Chromosome length is indicated in megabases on the left, and gene names are shown at their corresponding positions. *CgGATA23* was assigned to an unanchored genomic sequence (ChrUn).

**Figure 2:**
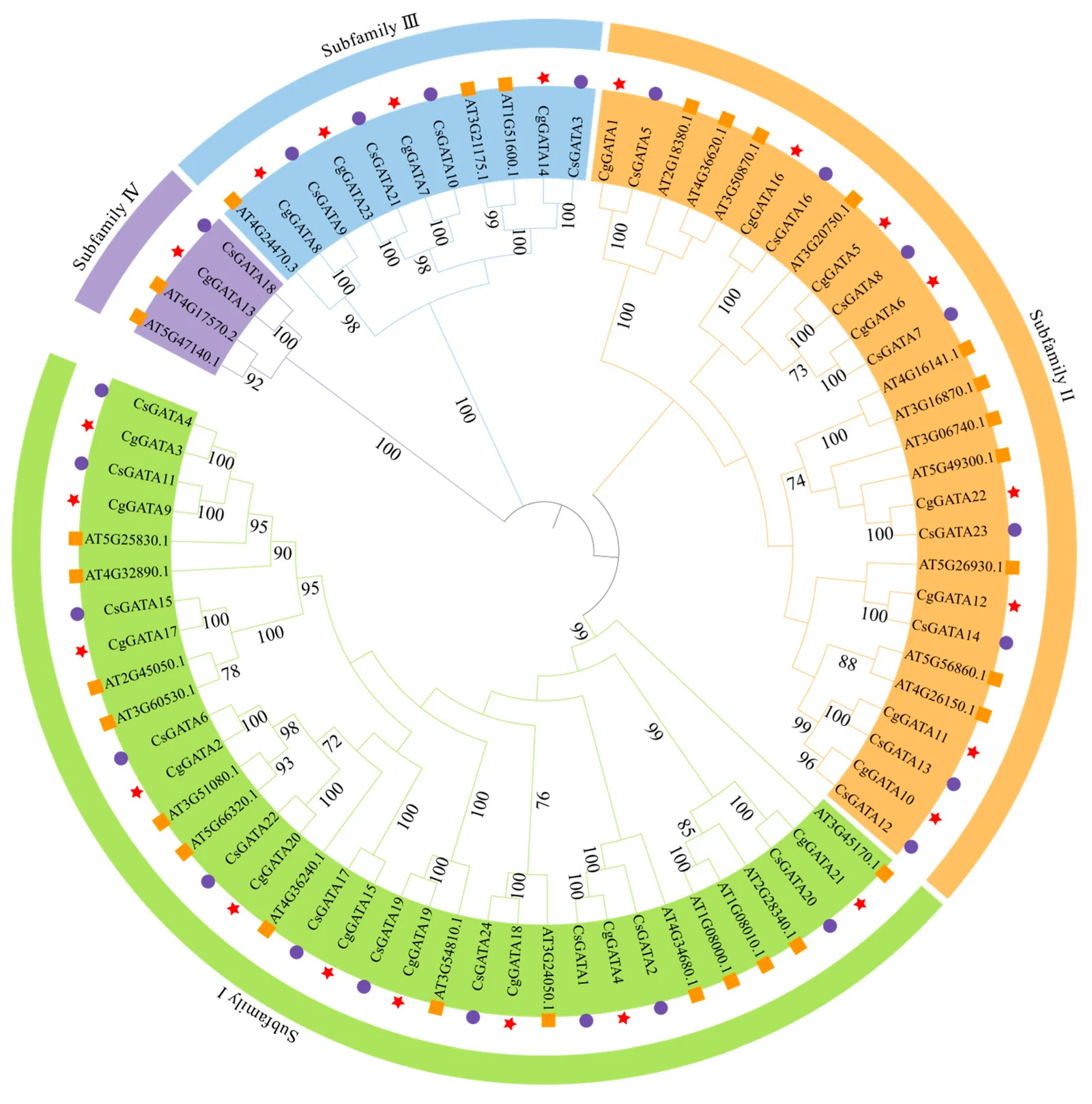
Neighbour-joining phylogenetic tree of GATA proteins from pummelo, sweet orange, and *Arabidopsis thaliana*. The four subfamilies (I–IV) are distinguished by background colour. CgGATA, CsGATA and AtGATA protein identifiers are shown at the terminal branches. Purple circles represent sweet orange (*Citrus sinensis*), red asterisks represent ‘Wanbai pummelo’ (*Citrus grandis* ‘Wanbai’), and orange squares represent *Arabidopsis thaliana*. Numbers at the nodes indicate bootstrap support from 1000 replicates.

**Figure 3:**
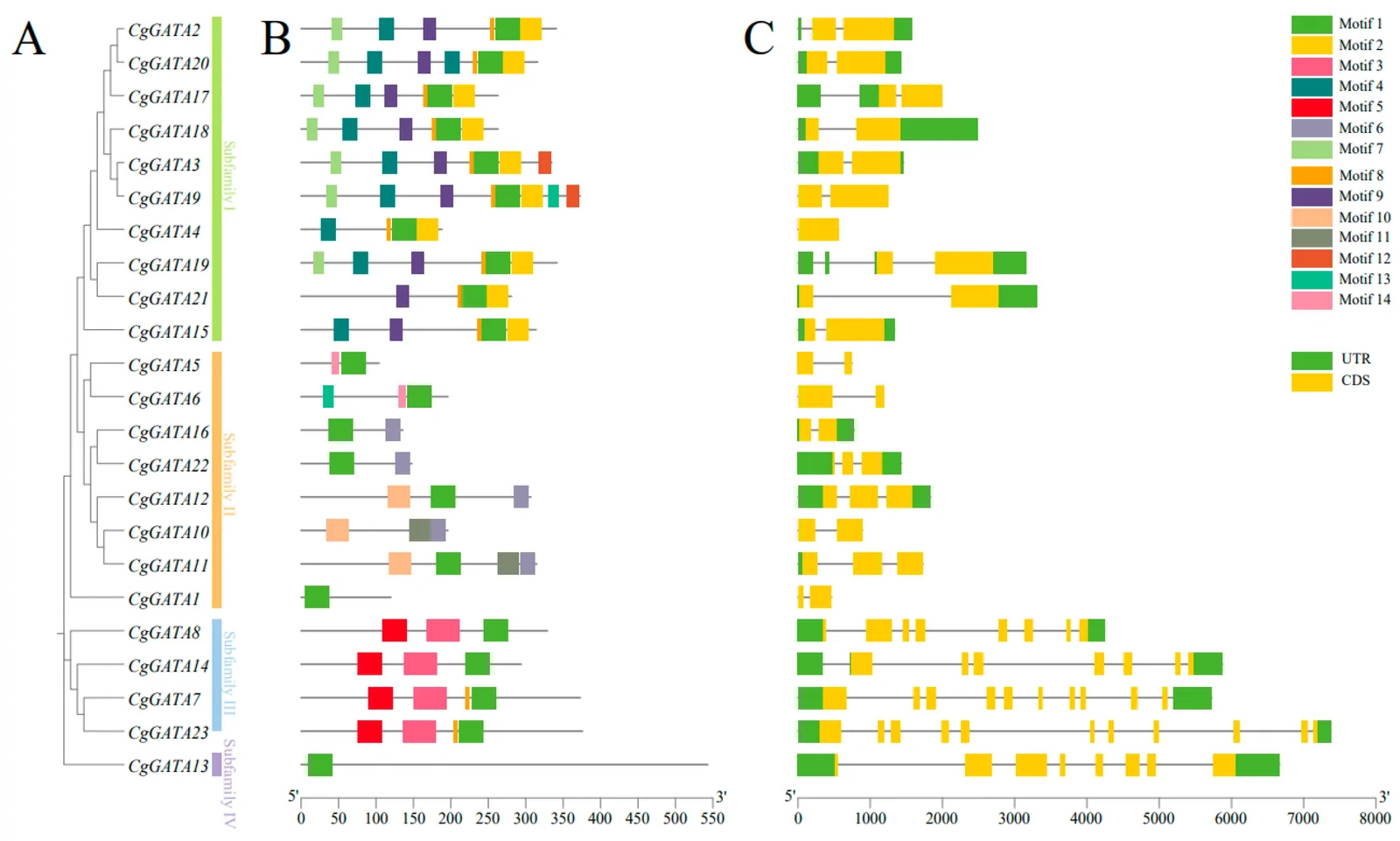
Phylogenetic relationships, conserved-motif distribution and gene structures of the *CgGATA* family. (**A**) Neighbour-joining phylogenetic tree of the 23 CgGATA proteins. (**B**) Distribution of conserved motifs identified by MEME; coloured boxes represent Motifs 1–14. (**C**) Exon–intron structures of the corresponding *CgGATA* genes. Green boxes indicate untranslated regions, yellow boxes indicate coding sequences, and grey lines indicate introns.

**Figure 4:**
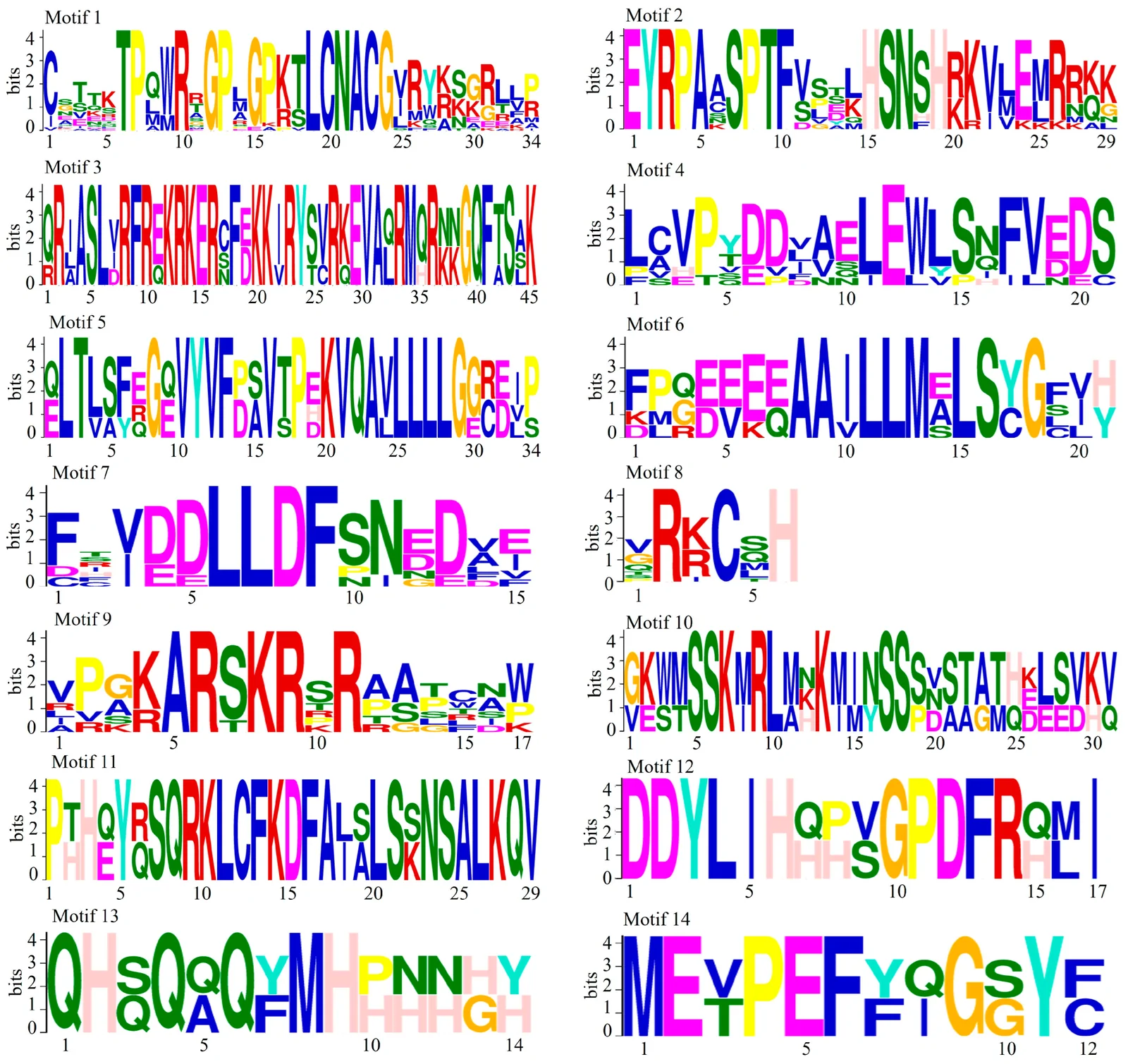
Sequence logos of the 14 conserved motifs identified in CgGATA proteins by MEME analysis. The height of each amino-acid symbol represents its relative conservation at that position. Motif 1 contains the conserved GATA zinc-finger DNA-binding domain characteristic of plant GATA transcription factors.

**Figure 5:**
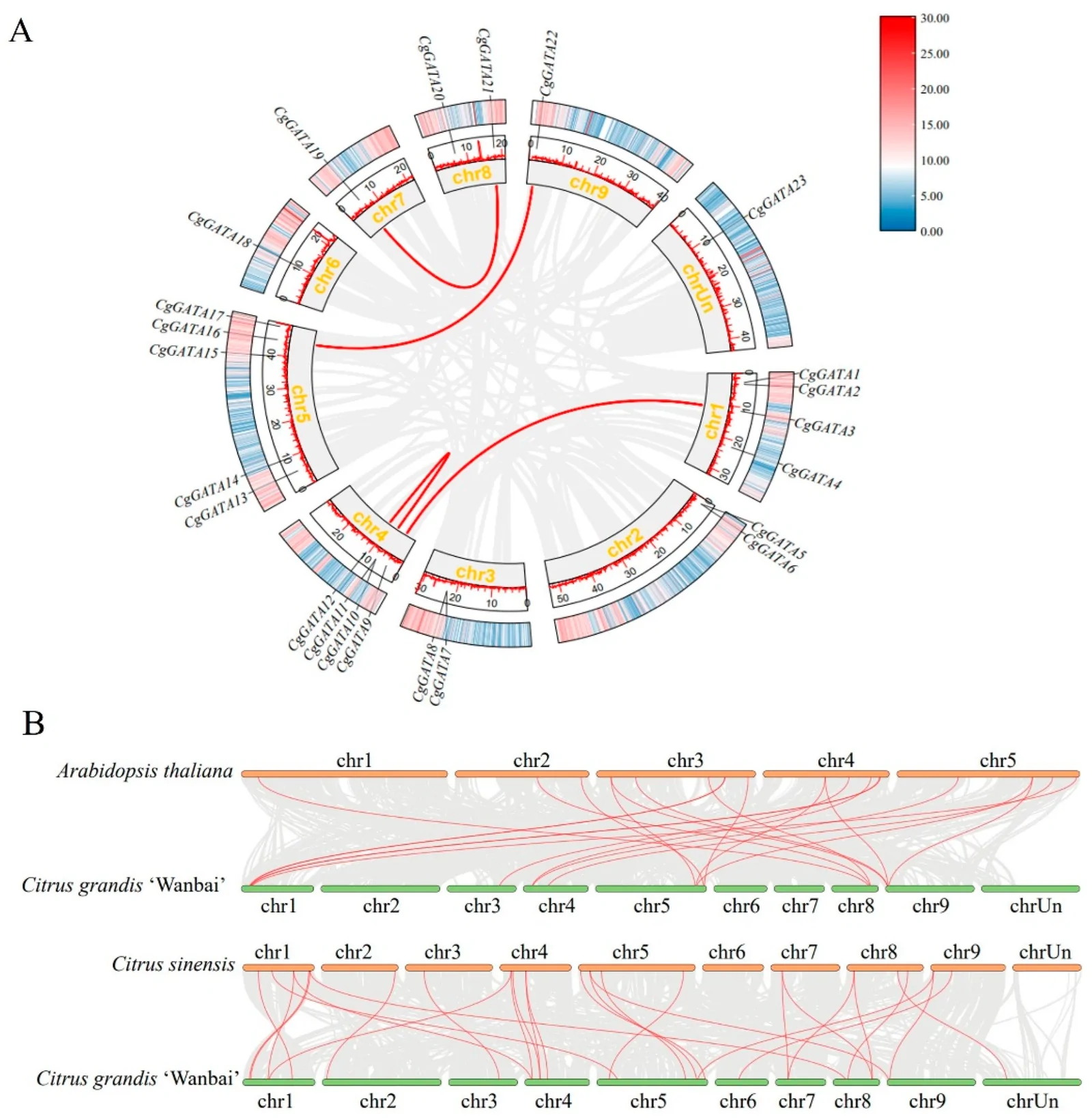
Intraspecific and interspecific collinearity of *GATA* genes. (**A**) Collinear relationships involving *GATA* genes within the pummelo genome. Grey lines represent genome-wide collinear blocks, and red lines indicate collinear pairs involving *CgGATA* genes. (**B**) Collinear relationships between pummelo and *Arabidopsis thaliana* and between pummelo and sweet orange. Grey lines represent genome-wide collinear blocks, and red lines indicate syntenic relationships involving *GATA* genes.

**Figure 6:**
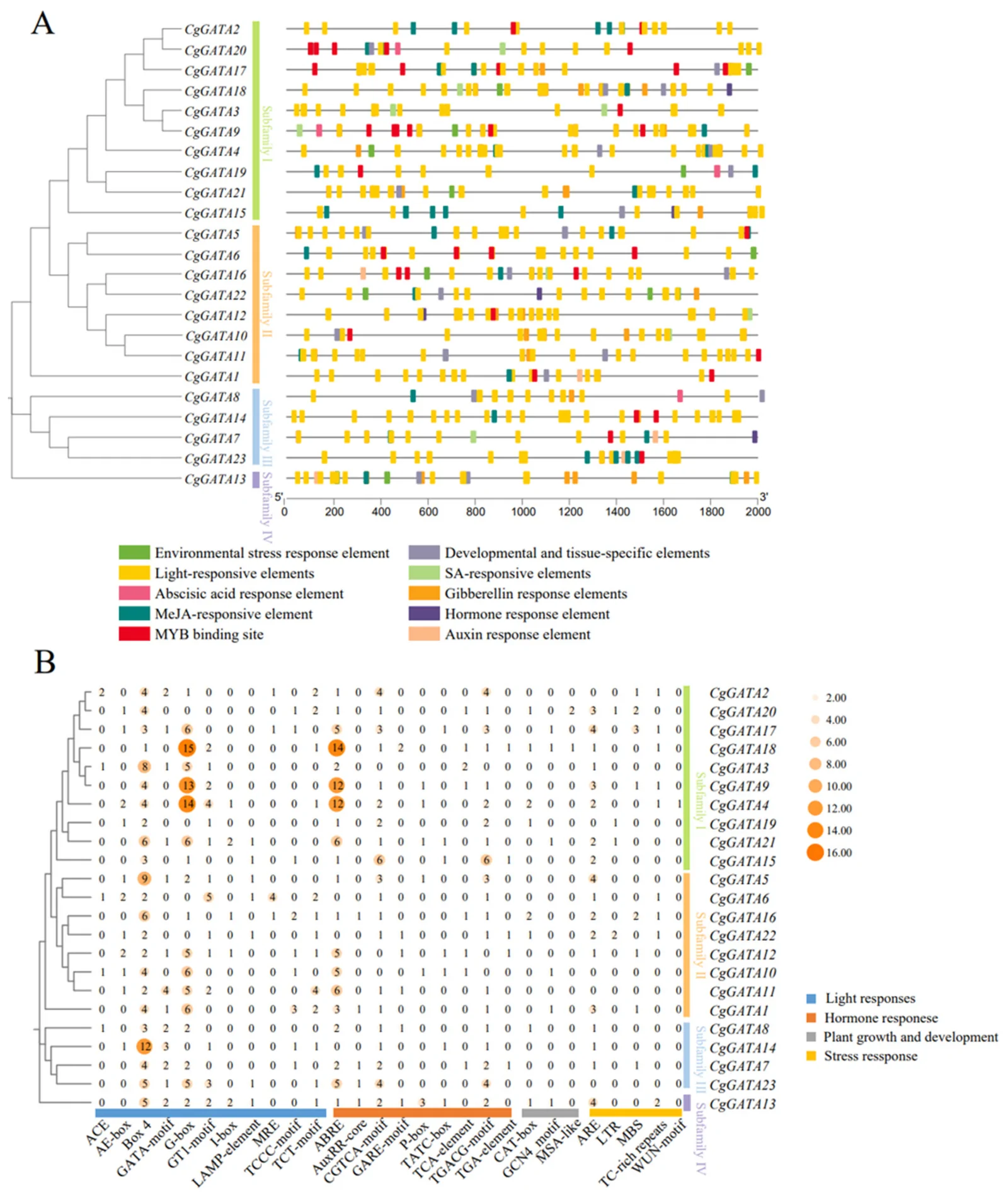
Predicted cis-acting elements in the putative upstream regulatory regions of *CgGATA* genes. (**A**) Distribution of selected elements within the 2000 bp sequences upstream of the annotated start codons. Element categories are represented by distinct colours. (**B**) Numbers of predicted elements in the light-response, phytohormone-response, growth-and-development, and stress-response categories. Circle size represents the number of predicted elements.

**Figure 7:**
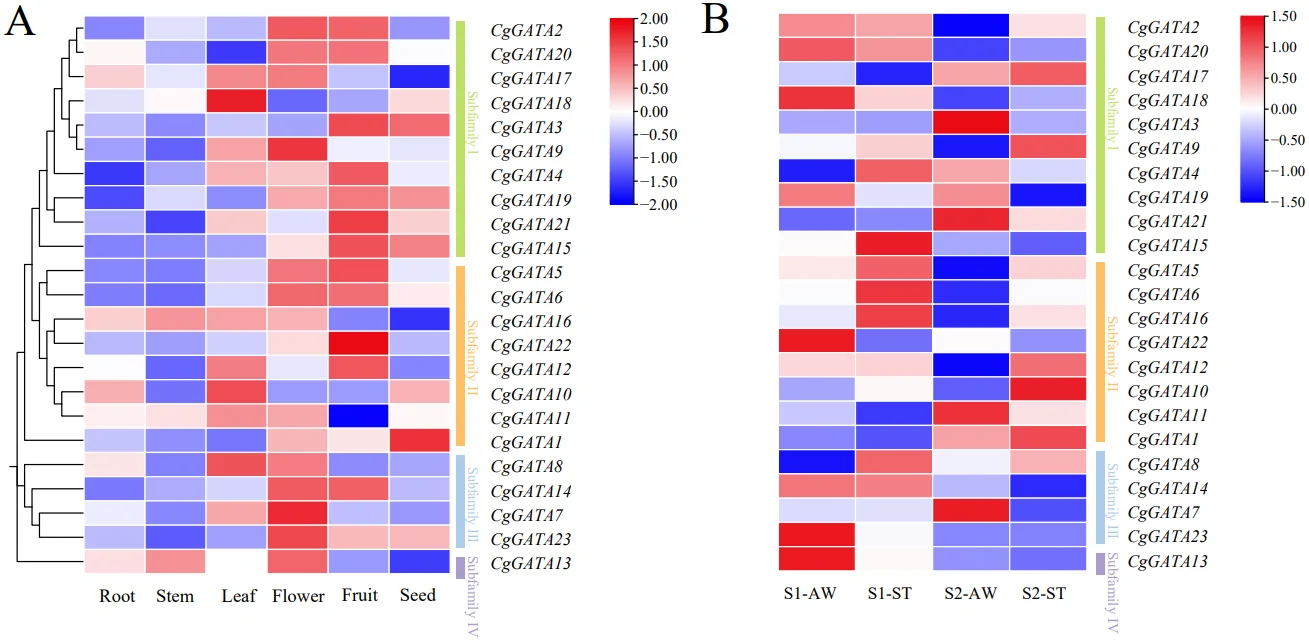
Expression profiles of *CgGATA* genes across sampled pummelo tissues and in contrasting plant-height genotypes. (**A**) Relative transcript abundance in roots, stems, leaves, floral tissue, fruits and seeds of ‘Wanbai Pummelo’. (**B**) Relative transcript abundance in spring-shoot tips of tall ‘Shatian Pummelo’ (ST) and dwarf ‘Aiwan Pummelo’ (AW) at S1 and S2. Heatmaps show row-wise-scaled relative expression values; red and blue indicate higher and lower values, respectively, within each gene. Colours do not indicate absolute expression differences between genes or statistical significance.

**Figure 8:**
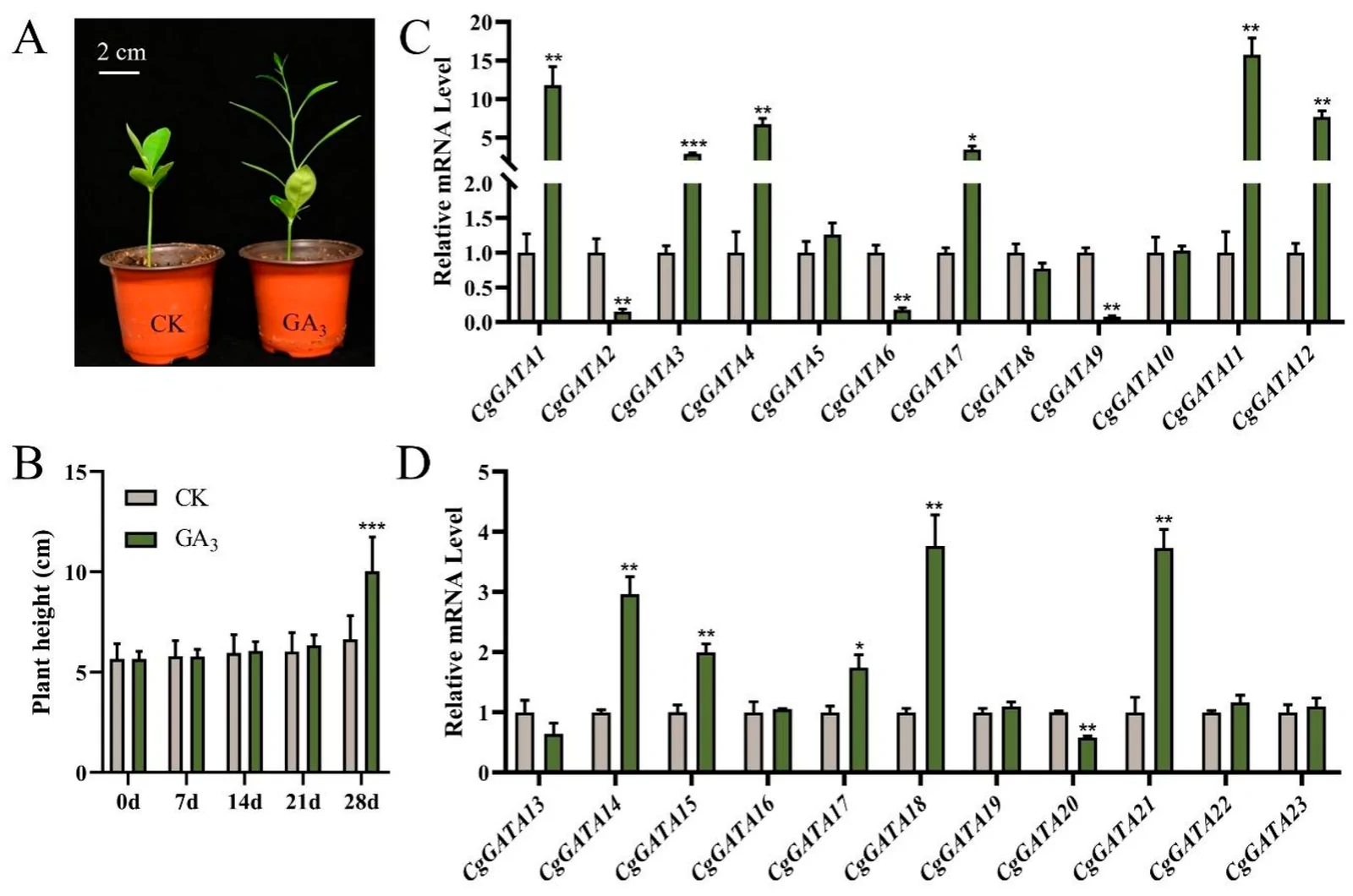
Effects of prolonged GA_3_ treatment on seedling growth and *CgGATA* transcript abundance. (**A**) Representative control and GA_3_-treated ‘Wanbai Pummelo’ seedlings at 28 d. Scale bar = 2 cm. (**B**) Plant height at 0, 7, 14, 21 and 28 d. Values represent the mean ± standard error (SE) of three biological replicates; each replicate contained 10 seedlings. A linear mixed-effects model was used to assess the effects of treatment, time, and their interaction, followed by post hoc comparisons as described in Section 4.2. (**C**,**D**) Relative transcript abundance of the 23 *CgGATA* genes in shoot-tip tissues collected at 28 d. Values represent the mean ± SE of three biological replicates. Two-sided Student’s *t*-tests were used for the gene-wise GA3-versus-control comparisons, as described in Section 4.8. Asterisks denote the significance levels shown in the figure (* *p* < 0.05, ** *p* < 0.01 and *** *p* < 0.001).

**Table 1:** Predicted physicochemical properties and subcellular localisation of CgGATA proteins in ‘Wanbai Pummelo’.

Gene	Sequence ID	Amino-Acid Length	Molecular Mass (kDa)	Theoretical pI	Instability Index	Aliphatic Index	GRAVY	Predicted Subcellular Localisation
*CgGATA1*	Cg1g004300.1	119	13.26	6.81	58.03	64.79	−0.452	Nucleus
*CgGATA2*	Cg1g005110.1	340	37.21	7.99	62.12	66	−0.584	Nucleus
*CgGATA3*	Cg1g014090.1	334	36.67	5.72	49.22	54.61	−0.609	Nucleus
*CgGATA4*	Cg1g022820.1	187	21.13	9.12	53.59	62.09	−0.699	Nucleus
*CgGATA5*	Cg2g001630.1	103	11.55	9.54	65.79	45.63	−1.275	Nucleus
*CgGATA6*	Cg2g001640.1	195	21.22	9.48	49.94	47.23	−0.931	Nucleus
*CgGATA7*	Cg3g013840.1	372	40.63	4.68	48.95	66.59	−0.777	Nucleus
*CgGATA8*	Cg3g013850.1	328	35.23	5.36	50.42	59.51	−0.612	Nucleus
*CgGATA9*	Cg4g004250.1	372	41.16	6.21	48.82	55.59	−0.785	Nucleus
*CgGATA10*	Cg4g006680.1	195	21.60	9.34	45.83	61.59	−0.768	Nucleus
*CgGATA11*	Cg4g006690.1	314	35.08	9.61	48.06	61.24	−0.782	Nucleus
*CgGATA12*	Cg4g009200.1	306	33.90	9.45	61.79	56.83	−0.855	Nucleus
*CgGATA13*	Cg5g007460.1	542	60.30	6.08	55.79	69.23	−0.655	Nucleus
*CgGATA14*	Cg5g012570.1	293	32.05	6.15	34.51	65.56	−0.787	Nucleus
*CgGATA15*	Cg5g031000.1	313	34.37	6.66	66.78	63.61	−0.721	Nucleus
*CgGATA16*	Cg5g037960.1	135	14.90	10.3	58.43	60.15	−0.69	Nucleus
*CgGATA17*	Cg5g043880.1	262	29.23	5.98	62.37	54.01	−0.9	Nucleus
*CgGATA18*	Cg6g007390.1	262	29.01	8.44	46.74	69.5	−0.589	Nucleus
*CgGATA19*	Cg7g006680.1	341	37.43	6.1	56.93	62.29	−0.613	Nucleus
*CgGATA20*	Cg8g008590.1	315	35.01	5.54	57.13	51.9	−0.693	Nucleus
*CgGATA21*	Cg8g020120.1	280	31.52	8.96	67.07	59.5	−0.754	Nucleus
*CgGATA22*	Cg9g001700.1	147	15.94	9.69	55.97	63.06	−0.839	Nucleus
*CgGATA23*	CgUng003830.1	375	41.16	4.77	49.85	60.16	−0.793	Nucleus

## Data Availability

The original contributions presented in this study are included in the article/Supplementary Material. Further inquiries can be directed to the corresponding authors.

## Supplementary Materials

The following supporting information can be downloaded at: https://www.mdpi.com/article/10.3390/ijms27198542/s1.
