# Supplementary material for: Genome-Wide Identification and Expression Profiling of the GATA Gene Family Reveals Candidate Genes Associated with GA3-Induced Shoot Elongation in Citrus grandis ‘Wanbai’
Source: Int J Mol Sci. 2026 Sep 24;27(19):8542. doi: 10.3390/ijms27198542 (PMC13665233; doi:10.3390/ijms27198542)
Supplement: Supplementary file 1 [file ijms-27-08542-s001.zip › Supplementary_Material_Revised.pdf]

Supplementary Material

Genome-Wide Identification and Expression Profiling of the GATA Gene Family Reveals Candidate Genes Associated with GA_3-Induced Shoot Elongation in Citrus grandis 'Wanbai'

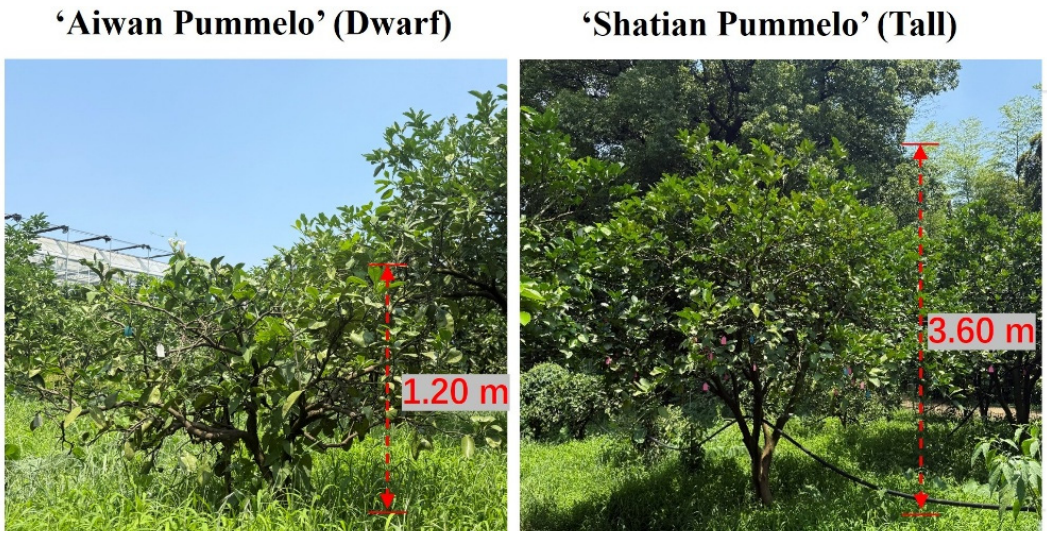

Figure S1. Representative phenotypes of the dwarf 'Aiwan Pummelo' and tall 'Shatian Pummelo'. The annotated arrows indicate the approximate tree heights shown in the photographs (1.20 and 3.60 m, respectively).

Table S2. Predicted GARE motifs in the 2,000-bp putative upstream regulatory regions of CgGATA11 and CgGATA18.

**Gene name**	**Motif type**	**Sequence**	**Position in extracted sequence**	**Length (bp)**	**Strand**	**Species annotation**	**Function**
CgGATA11	GARE-motif	TCTGTTG	1017	7	+	*Brassica oleracea*	gibberellin-responsive element
CgGATA18	GARE-motif	TCTGTTG	1238	7	-	*Brassica oleracea*	gibberellin-responsive element
CgGATA18	GARE-motif	TCTGTTG	1509	7	+	*Brassica oleracea*	gibberellin-responsive element

Note: The analysed sequence for each gene comprised 2,000 bp upstream of the annotated translation start codon (ATG). Strand denotes motif orientation within the submitted upstream sequence.

Table S3. Primer sequences used for RT-qPCR analysis.

Gene name	Forward primer (5′–3′)	Reverse primer (5′–3′)
β-Actin	CCGACCGTATGAGCAAGGAAA	TTCCTGTGGACAATGGATGGA
CgGATA1	GCTAACTGTGACACCACTTCTAC	GCCCGTATCAGAAGTTTGATC
CgGATA2	TATCAGTTGGGGTCAGTTTACTCG	GCCGCATCTCCATCACTTTAC
CgGATA3	CGATGACTTGGCAGAGTTAGAATG	CGGGTTGACGATGAAGAGGA
CgGATA4	CAGTGACACTAGCCACATAGC	CTGCTCGGGTGGCATAAC
CgGATA5	GATTACACGCTGCTCGAAG	CTACGCCACATTGGAGTATC
CgGATA6	CACTACTGCAAGCTCGTCAGC	TGCCTTCCTTTTCTCCTCCT
CgGATA7	GAAAGAGGTGGCACAGAGGAT	CGGACAACAGTTTCTGGACG
CgGATA8	AAGCGGTGCTGTTATTGTTGG	GCTGCTCTTTGTGGTTGGGTA
CgGATA9	GACCATTTCATTGTTGAGGAGC	GAGAAATTACGGCACCCGTTA
CgGATA10	GTCTCAACCTAAGACGATGACC	CTGATTCATCATGGGTTCCTG
CgGATA11	CACTCTTCGTCACATAATCGAG	GAACCGTTTTCGCTTGTAG
CgGATA12	TCAGAAGCAACCACCACCAT	CCTCTAGGTCCACTCCTCCAA
CgGATA13	AATTTGGGAGTGCAGATGCTAG	CTGGAGATTGCTTTGGACGAT
CgGATA14	ACTCCACAACGGTTGAGTGTCC	CATCCGAAGTGCTACTTCTTTACG
CgGATA15	GGGCAAGGCAAGAAGCAA	GGGTCGTCGGAGTGAAAGAGT
CgGATA16	ACGACAAGAACGCCTCTTTGG	CGCTTCTGTGCTCTTCCTTTG
CgGATA17	GACTCCTTTAATCCCTCCTCTGA	TCGCTTTTGAACGCTTGCT
CgGATA18	GCTTGTGGGGTTCGGTTTA	CATTTGCTTCTGTCTCCTCATCTC
CgGATA19	ACAGCTCCACTGACCTGTCCA	GGTTGTGAGATGAATCGTCCTTAG
CgGATA20	GGTCGTCTGGGTCACCGTTAT	TTTGACAGTGGCTACACCTCCT
CgGATA21	CGGTGGTGCCGACAAGTAA	AGGATTAATGGGTACGTGTTTATC
CgGATA22	GTCACTGTGTAATGCATGCG	GCTTATTACTTGAGCTATTACTGC
CgGATA23	GCGATGAATCCACAACCACTA	CCCTGTTTGCTGCCGTTT

Table S4. Differential-expression analysis and multiple-testing results reported for CgGATA genes in the S1-ST, S2-AW and S2-ST groups.

Gene	Discovery	p-value	q-value	Discovery	p-value	q-value	Discovery	p-value	q-value	Discovery	p-value	q-value
		S1-AW			S1-ST			S2-AW			S2-ST	
CgGATA1	–	–	–	Yes	0.004846626	0.007633436	Yes	0.022370776	0.023489315	Yes	0.003243361	0.007633436
CgGATA2	–	–	–	Yes	0.00121377	0.00254892	No	0.35841826	0.25089278	No	0.35416548	0.25089278
CgGATA3	–	–	–	Yes	0.006485549	0.013619653	No	0.161996537	0.170096364	No	0.433708477	0.303595934
CgGATA4	–	–	–	Yes	0.00059806	0.00094194	Yes	0.00034196	0.00094194	Yes	0.00181164	0.00190222
CgGATA5	–	–	–	No	0.99423	1	No	0.91312	1	No	0.942321	1
CgGATA6	–	–	–	Yes	0.00264994	0.00139122	Yes	0.00026516	0.00027842	No	0.85956225	0.30084679
CgGATA7	–	–	–	Yes	0.00488	0.00442345	Yes	0.09570315	0.0334961	Yes	0.00842562	0.00442345
CgGATA8	–	–	–	Yes	0.01061365	0.01114433	Yes	0.0022074	0.00695331	Yes	0.00749989	0.01114433
CgGATA9	–	–	–	No	0.21880424	0.64625415	No	0.61548014	0.64625415	No	0.52719008	0.64625415
CgGATA10	–	–	–	Yes	0.0008	0.00126	Yes	0.0059131	0.00620876	Yes	0.00076825	0.00126
CgGATA11	–	–	–	Yes	0.04155	0.0436275	Yes	0.00933469	0.01960285	No	0.29973947	0.20981763
CgGATA12	–	–	–	Yes	0.00000232	0.00000244	No	0.99234058	0.3473192	Yes	0.00873839	0.00458765
CgGATA13	–	–	–	Yes	0.00166978	0.00525981	Yes	0.00559144	0.00880652	Yes	0.02306394	0.02421714
CgGATA14	–	–	–	Yes	0.00018	0.000189	Yes	0.06069608	0.02124363	Yes	0.00827317	0.00434341
CgGATA15	–	–	–	Yes	0.04595714	0.04681809	Yes	0.06688298	0.04681809	Yes	0.0091734	0.01926414
CgGATA16	–	–	–	No	0.07811627	0.08202208	Yes	0.00700203	0.01470426	No	0.38338855	0.26837199
CgGATA17	–	–	–	Yes	0.007789	0.01226768	Yes	0.03239666	0.03401649	Yes	0.00388612	0.01224128
CgGATA18	–	–	–	Yes	0.0000209	0.00004694	Yes	0.0000298	0.00004694	Yes	0.00004879	0.00005123
CgGATA19	–	–	–	Yes	0.10932	0.038262	Yes	0.01625423	0.00853347	Yes	0.0000761	0.0000799
CgGATA20	–	–	–	Yes	0.00165	0.00086625	No	0.49794969	0.17428239	Yes	0.00117065	0.00086625
CgGATA21	–	–	–	Yes	0.0000879	0.00027689	Yes	0.02607052	0.02737405	Yes	0.00047335	0.00074553
CgGATA22	–	–	–	Yes	0.116652	0.0408282	Yes	0.00120164	0.00126172	Yes	0.00310141	0.00162824
CgGATA23	–	–	–	Yes	0.0000359	0.0000565	Yes	0.0000041	0.0000129	Yes	0.00053643	0.00056325

Note: S1-AW served as the normalized control group. Differential expression analysis was performed by comparing the treatment groups against S1-AW. Consequently, no P values were calculated for the control group itself. Abbreviations: ST, 'Shatian Pummelo'; AW, 'Aiwan Pummelo'; S1, rapid-elongation stage; S2, shoot-maturation stage.
